# Reduced Maximum Pitch Elevation Predicts Silent Aspiration of Small Liquid Volumes in Stroke Patients

**DOI:** 10.3389/fneur.2017.00436

**Published:** 2017-08-25

**Authors:** Akila Theyyar Rajappa, Kristie R. Soriano, Courtney Ziemer, Michelle S. Troche, Jaime Bauer Malandraki, Georgia A. Malandraki

**Affiliations:** ^1^Department of Biobehavioral Sciences, Teachers College, Columbia University, New York, NY, United States; ^2^Department of Speech Pathology and Audiology, JFK Rehabilitation Institute, Edison, NJ, United States; ^3^Department of Speech, Language, and Hearing Sciences, Purdue University, West Lafayette, IN, United States

**Keywords:** stroke, dysphagia, respiratory aspiration, pitch elevation, swallowing

## Abstract

**Background and purpose:**

Preliminary evidence has shown that reduced ability to maximally raise vocal pitch correlates with the occurrence of aspiration (i.e., airway invasion by food or liquid). However, it is unclear if this simple task can be used as a reliable predictor of aspiration in stroke patients. Our aim was to examine whether maximum vocal pitch elevation predicted airway invasion and dysphagia in stroke.

**Methods:**

Forty-five consecutive stroke patients (<1 month poststroke) at a rehabilitation setting participated in a videofluoroscopic swallow study and two maximum vocal pitch elevation tasks. Maximum pitch was evaluated acoustically [maximum fundamental frequency (max *F*_0_)] and perceptually. Swallowing safety was rated using the Penetration/Aspiration Scale and swallowing performance was assessed using components of the Modified Barium Swallow Impairment Profile (MBSImPTM©). Data were analyzed using simple regression and receiver operating characteristics curves to test the sensitivity and specificity of max *F*_0_ in predicting aspiration. Correlations between max *F*_0_ and MBSImP variables were also examined.

**Results:**

Max *F*_0_ predicted silent aspiration of small liquid volumes with 80% sensitivity and 65% specificity (*p* = 0.023; area under the curve: 0.815; cutoff value of 359.03 Hz). Max *F*_0_ did not predict non-silent aspiration or penetration in this sample and did not significantly correlate with MBSImP variables. Furthermore, all participants who aspirated silently on small liquid volumes (11% of sample) had suffered cortical or subcortical lesions.

**Conclusion:**

In stroke patients (<1 month poststroke), reduced maximum pitch elevation predicts silent aspiration of small liquid volumes with high sensitivity and moderate specificity. Future large-scale studies focusing on further validating this finding and exploring the value of this simple and non-invasive tool as part of a dysphagia screening are warranted.

## Introduction

Oropharyngeal dysphagia is seen in more than 50% of patients post stroke (1), with 10–15% of stroke survivors experiencing persistent dysphagia for more than 6 months (2). Importantly, post stroke dysphagia may lead to malnutrition, dehydration, aspiration pneumonia, increased length of hospital stay, reduced quality of life, or death (1, 3). Therefore, early identification of dysphagia in this population is of paramount importance for preventing complications and improving patient outcomes (1).

Current best practice in dysphagia evaluation in stroke predominantly includes nurse-administered screenings (4–7) that then trigger a referral for a comprehensive assessment by a speech-language pathologist (SLP). Subsequently, SLPs perform clinical swallowing evaluations (CSEs) (8, 9) to determine whether an instrumental swallowing assessment [videofluoroscopic swallowing study (VFSS), or flexible endoscopic evaluation of swallowing (FEES)] is warranted. Despite their widespread clinical utility, the sensitivity and specificity of dysphagia screenings remains variable. In addition, intra- and inter-rater reliability for CSEs is relatively low (10, 11). Crucially, the incidence of undetected silent aspiration (i.e., aspiration without a cough response) on CSEs in neurologically impaired patients is reported to be as high as 42% (12), significantly impacting their rehabilitation potential. Thus, the need to identify additional clinical signs of aspiration and dysphagia in stroke is urgent in order to enhance early referral and treatment.

Maximum vocal pitch elevation and swallowing share several anatomical and neurophysiological substrates (13–17). Preliminary evidence has revealed that reduced ability to maximally raise vocal pitch correlates with the occurrence of aspiration (18). Specifically, in a study of 40 patients with dysphagia of variable etiologies, lower maximum pitch elevation measured acoustically and perceptually was associated with more severe airway invasion during swallowing [measured by the Penetration/Aspiration Scale (PAS)] (19). The authors hypothesized that lesions impacting the superior laryngeal nerve (SLN) and/or chronic aspiration resulting in diminished sensation, affected the sensorimotor processes required for both voice and swallowing in their sample. This study aimed to expand these findings (18) by focusing on stroke patients early post stroke (<1 month post) and improving several methodological limitations of the prior study.

Our primary aim was to determine if maximum pitch elevation of the sound /i/ and/or /a/ (measured acoustically and perceptually) predicted aspiration and/or silent aspiration in stroke. Based on prior work in this area (18), we hypothesized that maximum pitch elevation would significantly predict these events. Second, we aimed to determine whether maximum pitch elevation correlated with ratings of laryngeal elevation, anterior hyoid excursion, and pharyngeal residue. We hypothesized that reduced maximum pitch elevation would correlate with more severe ratings of swallowing physiology.

## Materials and Methods

### Participants

This study was approved by the Institutional Review Boards of the University and hospital that participated. Written informed consent was obtained from all participants in accordance with the Declaration of Helsinki. Inclusion criteria were as follows: diagnosis of stroke, referral for a VFSS because of probable dysphagia, and being alert and able to follow simple commands (e.g., “raise your pitch while producing the sound /i/ and /a/”). Exclusion criteria were as follows: reduced alertness, difficulty following simple commands, active respiratory disease, tracheostomy, history of formal vocal training, active smoking, pregnancy, and/or being medically fragile. Patients were recruited consecutively over a 6-month period (June 2015 to December 2015) from the stroke unit of a rehabilitation hospital.

### Data Collection

#### Stroke-Specific Information/Characteristics

Stroke-specific factors were collected by two trained research assistants (certified SLPs) by reviewing Neurology reports of patients’ medical charts. Age, sex, time of onset, type of stroke, and side and site of lesion were recorded from the review. The research assistants were blinded to swallowing and voice evaluation results. Stroke-specific factors were documented in patients’ medical charts by the treating neurologists.

#### Voice Tasks and Recordings

In order to determine the best methodology for using pitch elevation for swallowing screening, we examined differences between two vowels (/a/ and /i/) in achieving maximum pitch elevation. Both /a/ and /i/ are frequently used in clinical practice and there is evidence that the vowel /i/ may help individuals achieve higher pitch values (20). To obtain these values, first, patients were instructed to perform a maximum pitch elevation task using the vowels /i/ and /a/ during simultaneous videofluoroscopic recording. Following each trial, participants were asked to rate their own perception of vocal pitch on a 5-point visual analog scale (5 = highest possible pitch; 1 = not at all their highest). This scale was used to encourage participants to reach their highest pitch and confirm production of self-perceived highest pitch. Prior to data collection, patients participated in two practice trials per vowel after clinician modeling. Following the protocol of Malandraki and colleagues (18), patients were instructed to take a deep breath and begin saying /i/ or /a/ in their normal voice and slowly glide their voice as high in pitch as possible, holding that maximum pitch for 3–4 s. Participants were also advised not to move their head, trunk, or body during productions. This was repeated three times for each vowel. Voice recordings were obtained using a digital voice recorder (Tascam, DR-40, Linear PCM recorder) that was placed 15 cm from the participant’s mouth. Input level was set at 60 dB SPL. Frequency settings were set at 44.1–48 kHz/32 bits/s.

#### Swallowing Tasks and Recordings

Videofluoroscopic swallow evaluations were completed in a radiology suite using a GE Digital Fluoroscopy Unit (GE Medical System), which acquired images at full resolution (30 pulses/s). Images were recorded at 30 frames/s. Participants were imaged in an upright-seated position in lateral view to allow visualization of the lips, nasal cavity, cervical vertebrae, and the pharyngoesophageal segment. Bolus trials were standardized and conformed partially to the Modified Barium Swallow Impairment Profile (MBSImP) protocol (21). Participants completed: 5 mL (twice, clinician fed by spoon), 10 mL, and 40 mL of Varibar Thin Liquid (EZ-EM#D105, self fed by cup), and 5 mL Varibar Pudding (EZ-EM#125, clinician fed by spoon).

### Data Analysis

Voice analysis was performed using methods previously described (18). In short, audio files were de-identified using the Audacity software (22). To determine reliability between acoustic and perceptual voice analysis methods used to evaluate maximum pitch, we performed both types of analyses and explored correlations between the two measurement types. Acoustic analysis (Version 5.3.53) was performed by a trained certified SLP using Praat (23). Maximum fundamental frequency (max *F*_0_, Hertz) was the acoustic variable of interest (18).

Perceptual voice analysis was performed by a certified SLP and Board Certified Specialist in swallowing and swallowing disorders. The rater was blinded to patient diagnosis and swallowing performance. Participant’s age and sex were provided to this clinician to facilitate accurate maximum pitch judgments. A binary scale was devised inclusive of choices “Normal” and “Abnormal.” The clinician was instructed to listen to the trials of each vowel and rate their perception of the participant’s maximum pitch elevation ability using the binary scale (18). They were also instructed not to rate any other voice components, e.g., voice quality, hoarseness, or loudness. Ten percent of samples were re-analyzed for reliability.

Videofluoroscopic analysis was performed using the 8-point PAS (19) and selected MBSImP components (21). The 8-point PAS is an equal appearing interval scale to describe penetration and aspiration events (19). Penetration is defined as passage of material (foods or liquids) into the larynx that does not pass below the level of the true vocal folds. Aspiration is defined as passage of material (foods or liquids) into the larynx that passes below the level of vocal folds. Scores are determined by (a) the depth to which material passes in the airway and (b) by whether or not material entering the airway is expelled (19). For example, a PAS score of 1 (lowest score) indicates material not entering the airway, whereas a PAS score of 8 (highest score) indicates material entering the airway below the vocal folds with no effort to expel the material, i.e., silent aspiration (see Table SA in Supplementary Material for detailed description of the scale and cutoff points/definitions used for analysis). Regarding swallowing physiology, swallows were analyzed for the following MBSImP components: laryngeal elevation, anterior hyoid excursion, and pharyngeal residue (21). Laryngeal elevation was rated using a 4-point scale, where 0 indicates superior movement of thyroid cartilage that results in complete approximation of the arytenoids to the epiglottic petiole, and 3 indicates no superior movement of thyroid (21). Anterior hyoid excursion was rated using a 3-point scale, where 0 indicates complete anterior hyoid movement and 2 indicates no anterior movement of hyoid (21). Pharyngeal residue was rated using a 5-point scale, where 0 indicates complete pharyngeal clearance and 4 indicates minimal to no pharyngeal clearance (21). For analysis purposes, swallows were grouped into three categories: small liquid boluses (5 and 10 mL trials), large liquid bolus (40 mL trial), and solid bolus (5 mL pudding). VFSS analysis was performed by an SLP certified in MBSImP. The clinician was blinded to patient identity and diagnosis. Ten percent of all swallows were re-analyzed for reliability.

### Statistical Analysis

Statistical analysis was conducted using SPSS (Version 22, Armonk, NY, USA). Voice variables considered for analysis were max *F*_0_ and clinician perceptual pitch ratings. Swallowing variables included worse/maximum PAS scores and MBSImP component scores for each bolus category. Inter-rater and intra-rater reliability for acoustic/perceptual and videofluoroscopic data was calculated using Cohen’s weighted kappa (24). Pearson correlations were used to examine correlations between the two vowels, and independent *t*-tests were used to compare mean max *F*_0_ values between patients with normal and abnormal maximum pitch perception ratings, as a measure of reliability between the two voice evaluation methods.

To investigate the main aims of the study, simple regression analysis was used to evaluate whether max *F*_0_ influenced PAS scores for each bolus category, when age-, sex-, and disease-specific factors were also considered. Receiver operating characteristics (ROC) curves were plotted to test the sensitivity and specificity of max *F*_0_ in predicting aspiration. ROC analysis compared: (1) participants with normal swallowing with those with penetration-aspiration on VFSS (PAS scores 1–2 vs. 3–8), (2) participants with normal to mild changes in swallowing to those with aspiration on VFSS (PAS scores 1–5 vs. 6–8), (3) participants with at least penetration to those who aspirated on VFSS (PAS 3–5 vs. 6–8), and (4) participants who did not exhibit silent aspiration on VFSS to those who had silent aspiration (PAS 1–7 vs. 8) (see Table SA in Supplementary Material for cutoff points’ definitions). The sensitivity and specificity with the optimal cutoff value were derived from the analysis as a measure of validity. ROC curves and the corresponding areas under the curve (AUC) were calculated for max *F*_0_. A *p*-value of 0.05 was regarded as statistically significant. Finally, Pearson correlations were used to assess the relationship between acoustic/perceptual and MBSImP component measures.

## Results

### Demographic Data

A total of 72 consecutive patients (39 males), referred by their physicians for a swallowing evaluation as part of their routine clinical care, were screened. Twenty-seven patients were excluded; five patients had suffered a stroke more than 1 month prior to the study, eight had a non-stroke diagnosis, seven had other concomitant non-neurogenic diagnoses (e.g., head/neck cancer), and seven were excluded due to difficulty completing the procedures, previous voice or musical training, and/or technical/equipment failures. Analysis was completed on the remaining 45 patients (22 males), who had suffered a stroke 2–25 days prior to participation. Age range was 43–94 years (mean: 71.5 years). Aggregate demographic and stroke-specific characteristics (time of onset, type, and site of lesion) are reported in Table 1. Detailed data for each participant are presented in Table SB in Supplementary Material.

**Table 1:** Participant demographic and stroke-specific characteristics (*n* = 45).

Variable	*N*	%
**Sex**		
Male	22	49
Female	23	51
Age (years and months)	Mean (SD): 71.56 (13.58) Range: 43–94	
**Primary side of current lesion (CVA)**		
Right	25	56
Left	12	27
Bilateral	8	17
Onset (days prior to evaluation)	Mean (SD): 11.42 (5.82) Range: 2–25	
**Type of stroke**		
Ischemic	32	71
Hemorrhagic	13	29
**Site/s of lesion**		
Cortical	20	45
Subcortical	9	20
Brainstem	5	11
Multiple	11	24

### Reliability

There was substantial to excellent inter- and intra-rater agreement for the VFSS measures of laryngeal elevation (Cohen’s κ_w_ = 0.683, *p* = 0.009; and Cohen’s κ_w_ = 0.843, *p* = 0.002, respectively), anterior hyoid excursion (Cohen’s κ_w_ = 0.639, *p* = 0.001; and Cohen’s κ_w_ = 0.755, *p* = 0.005, respectively), pharyngeal residue (Cohen’s κ_w_ = 0.806, *p* = 0.003; and Cohen’s κ_w_ = 0.831, *p* = 0.002, respectively), and PAS scores (Cohen’s κ_w_ = 0.876, *p* < 0.001; and Cohen’s κ_w_ = 0.876, *p* < 0.001, respectively). Clinician perceptual pitch ratings revealed excellent inter- and intra-rater agreement (Cohen’s κ_w_ = 0.837, *p* = 0.001; and Cohen’s κ_w_ = 0.80, *p* = 0.010, respectively).

### Determining Best Methodology for Evaluating Maximum Pitch-Correlations between Vowels /i/ and /a/

Results revealed excellent correlation between the max *F*_0_ of the two vowels (*r* = 0.927, *p* < 0.001) with the vowel /i/ exhibiting less variance. There was also excellent correlation between their perceptual ratings (*r* = 0.911, *p* < 0.001). Therefore, the vowel /i/ was used for further analyses.

### Relationship between Acoustic Measures and Perceptual Pitch Ratings

Participants who were clinically rated as having abnormal pitch elevation skills had significantly decreased max *F*_0_ (mean = 286.22 Hz, SD = 77.41, SE = 16.5) compared to participants who were rated as having normal maximum pitch elevation skills (mean = 518.45 Hz, SD = 78.48, SE = 16.36) (*p* < 0.001). Therefore, the acoustic measure of max *F*_0_ was used for the remaining analyses, as a more accurate measure of vocal pitch.

### Relationship between PAS Scores and Max *F*_0_

**Figure 1:**
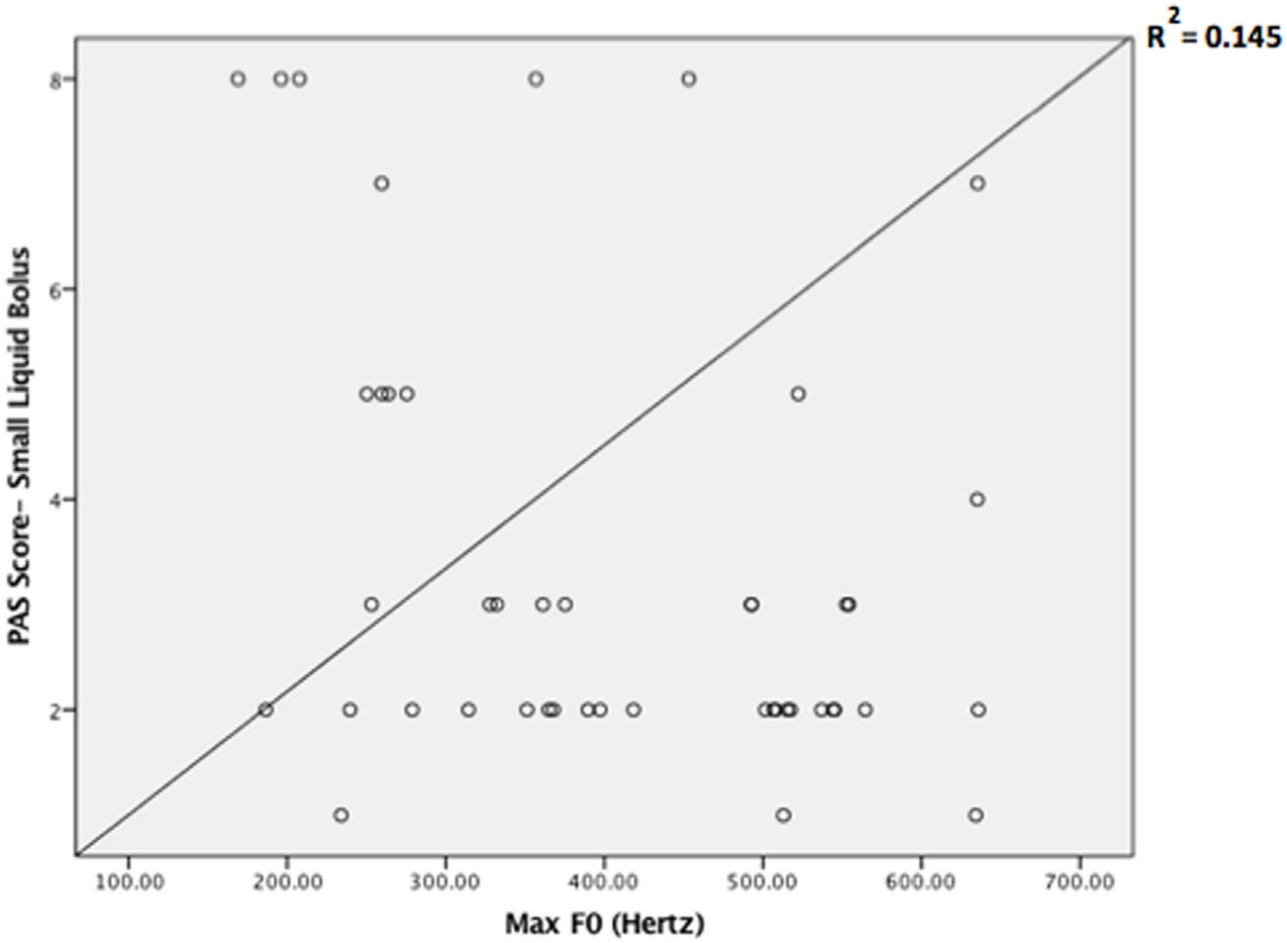
Scatter plot of the Penetration/Aspiration Scale (PAS) scores for small liquid bolus (5–10 mL) across maximum fundamental frequency (max *F*_0_) values.

**Figure 2:**
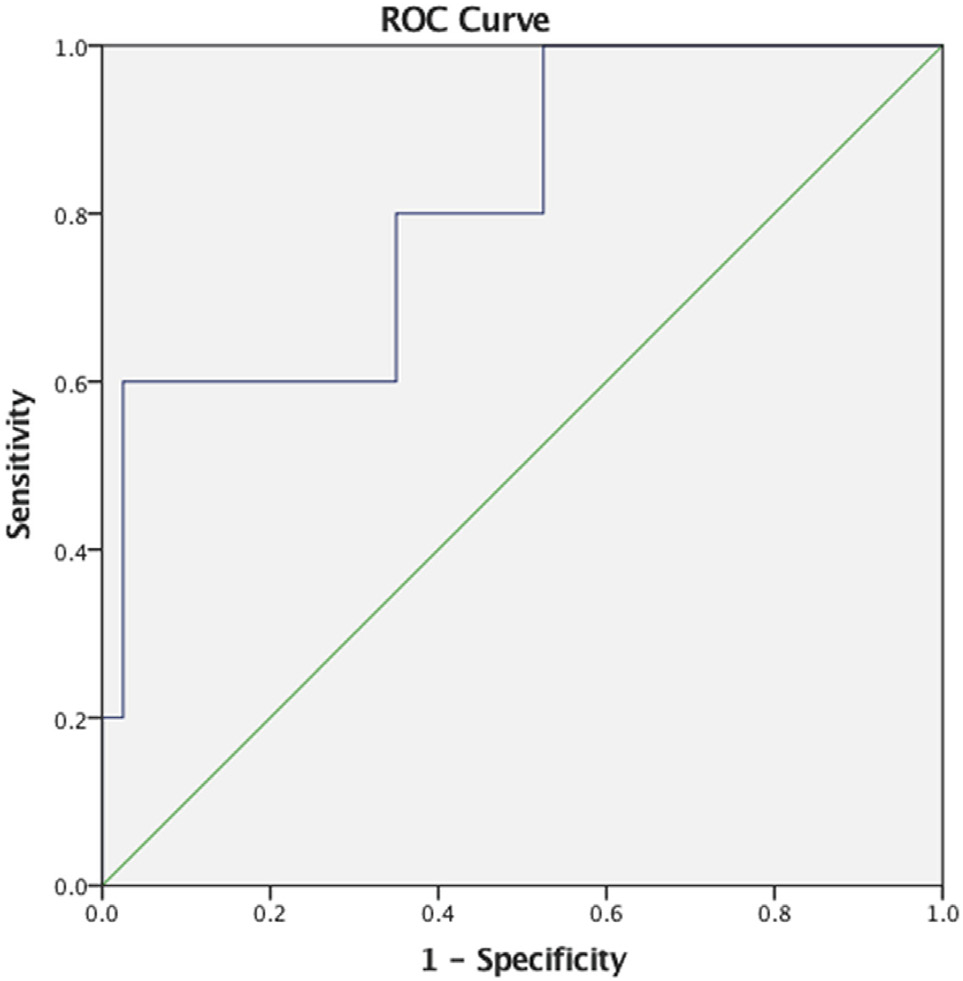
Receiver operating characteristics (ROC) curve for maximum fundamental frequency as a predictor of small liquid bolus silent aspiration (Penetration/Aspiration Scale score 8).

Descriptive statistics on max *F*_0_, PAS, and MBSImP components measures for the three-bolus types (5–10 mL, 40 mL thin liquids, and 5 mL pudding) are shown in Table 2. Regression analysis revealed that max *F*_0_ was the only significant factor influencing small bolus (5–10 mL) PAS scores when age-, sex-, and disease-specific factors (time of onset, site and side of lesion, and type of stroke) were accounted for [*F*_(1, 43)_ = 7.288, *p* < 0.001, with a *R*^2^ = 0.145261; Figure 1]. Therefore, we ran ROC curve analyses to assess the predictive value of max *F*_0_ for small bolus volume comparing the different categories of PAS scores (1–2 vs. 3–8; 1–5 vs. 6–8; 3–5 vs. 6–8; and 1–7 vs. 8). Max *F*_0_ significantly discriminated between patients with PAS scores 1–7 vs. 8 for the small liquid volumes (*p* = 0.023, AUC: 0.815, sensitivity 80%, specificity 65% for a cutoff value of 359.03 Hz; Figure 2), but did not significantly predict PAS scores for any other category (Table 3).

**Table 2:** Descriptive statistics on maximum fundamental frequency (max *F*_0_), Penetration/Aspiration Scale (PAS) scores and Modified Barium Swallow Impairment Profile (MBSImP) component scores for the three-bolus categories (5–10 mL, 40 mL thin liquids; 5 mL pudding) (*n* = 45).

Measures	Min	Max	Mean	SD
Max *F*_0_	169.04	635.79	406.48	139.08
**PAS (possible scores: 1–8)**				
5–10 mL	1	8	3.40	2.14
40 mL	1	8	4.02	2.30
Pudding (5 mL)	1	3	1.26	0.58
**MBSImP laryngeal elevation component (possible scores: 0–3)**				
5–10 mL	0	2	1.13	0.41
40 mL	1	2	1.11	0.32
Pudding (5 mL)	1	1	1.00	0.00
**MBSImP anterior hyoid excursion component (possible scores: 0–2)**				
5–10 mL	1	2	1.12	0.33
40 mL	0	2	1.09	0.34
Pudding (5 mL)	1	2	1.04	0.19
**MBSImP pharyngeal residue component (possible scores: 0–4)**				
5–10 mL	1	3	1.80	0.46
40 mL	1	2	1.86	0.35
Pudding (5 mL)	0	3	1.65	0.75

**Table 3:** Results from receiver operating characteristics curve (ROC) analyses for maximum fundamental frequency for small bolus (5–10 mL thin liquids) Penetration/Aspiration Scale (PAS) score category (*n* = 45).

PAS score category	Area under the curve	SE	Sig. *P*	Sens (%)	Spec (%)	Likelihood ratio (positive)	Likelihood ratio (negative)	Cutoff (Hz)
1–2 vs. 3–8	0.636	0.085	0.117	77.3	43.50	1.37	0.52	507.34
1–5 vs. 6–8	0.711	0.130	0.079	85.7	42.10	1.48	0.34	497.14
3–5 vs. 6–8	0.686	0.144	0.169	85.7	40.00	1.43	0.36	472.88
1–7 vs. 8	0.815	0.102	0.023*	80.0	65.00	2.33	0.28	359.03

*p < 0.05.

### Relationship between MBSImP Variables and Max *F*_0_

As seen in Table 4, there were no significant correlations between max *F*_0_ and most MBSImP components across bolus volumes (5–10 mL, 40 mL thin liquids, and 5 mL pudding; *p* > 0.05). Laryngeal elevation for 40 mL thin liquids was the only parameter that showed a significant but small negative correlation with max *F*_0_ (*r* = −0.327, *p* = 0.03) (Table 4).

**Table 4:** Correlations (Pearson *r* values) between maximum fundamental frequency (max *F*_0_) and Modified Barium Swallow Impairment Profile (MBSImP) component scores across bolus volumes (5–10 mL, 40 mL thin liquids, and 5 mL pudding) (*n* = 45).

Max *F*_0_			

MBSImP variables	5–10 mL	40 mL	5 mL pudding
Laryngeal elevation	−0.222 (*p* = 0.14)	−0.327 (*p* = 0.03)*	0.099 (*p* = 0.46)
Anterior hyoid excursion	0.003 (*p* = 0.985)	−0.252 (*p* = 0.09)	−0.064 (*p* = 0.69)
Pharyngeal residue	0.057 (*p* = 0.71)	0.003 (*p* = 0.99)	0.008 (*p* = 0.96)

*p < 0.0.5.

## Discussion

In the present study, we primarily aimed to determine if the simple task of maximally elevating vocal pitch was a reliable predictor of aspiration and/or silent aspiration in stroke patients. Results revealed that reduced maximum pitch elevation measured acoustically (max *F*_0_) significantly predicted silent aspiration for small liquid volumes (5–10 mL) with high sensitivity and moderate specificity.

Specifically, max *F*_0_ was successful in detecting the presence of silent aspiration with good discriminative ability, as evidenced by an AUC of 0.815 demonstrating high sensitivity (80%) and moderate specificity (65%), making it a potentially promising tool for screening in stroke settings, where timely referrals for VFSS/FEES is warranted. Although the number of silent aspirators for the small liquid volumes was relatively small (*n* = 5/45, 11%), max *F*_0_ was still able to effectively identify silent aspirators at a cutoff point of 359.03 Hz. Malandraki and colleagues (18) reported that patients in their sample who exhibited silent aspiration (*n* = 3) all had reduced max *F*_0_ (<387 Hz), but no ROC analysis was conducted. To our knowledge, this is the first study that offers a direct link between silent aspiration of small liquid volumes and reduced maximum pitch elevation in stroke patients. It is also important to note that max *F*_0_ was the only significant factor predicting small bolus PAS scores, after age-, sex-, and stroke-specific factors were accounted for.

These findings are in partial agreement with Malandraki et al. (18), who found that max *F*_0_ and perceptual pitch were associated with more severe PAS scores across bolus consistencies. Although the trials given to patients were similar to the present methodology, in that study they did not analyze small vs. large volumes separately and only tested the vowel /a/ on a heterogeneous group of patients. On the contrary, our study focused specifically on stroke patients early post stroke (<1 month post), included the vowel /i/ as a potentially better method of achieving maximum pitch, and confirmed highest maximum pitch ability with the subjects using a visual analog scale.

Pitch elevation and swallowing share anatomical and peripheral neurophysiological substrates that involve the internal and external branches of the SLN (iSLN and eSLN). The iSLN is a sensory branch of the vagus that plays an important role in laryngo-pharyngeal sensation (13, 14). The eSLN is a motor branch that plays a role in vocal pitch elevation, through innervation of the cricothyroid (15), and partially innervates the inferior pharyngeal constrictor enabling pharyngeal bolus clearance (16). Activation of the strap muscles and the cricopharyngeus, innervated primarily by fibers of the pharyngeal and cervical plexuses, is also observed in both pitch elevation and swallowing (17). Patients with dysphagia secondary to neurological insult, which may have impacted these shared substrates, may exhibit compromised performance in both functions.

In addition, we observed that all participants who aspirated silently on small liquid volumes (11% of sample) had suffered cortical or subcortical strokes. Laryngeal sensory stimuli are known to be processed differentially at the different levels of the neuraxis (brainstem vs. subcortex/cortex), with larger/stronger stimuli being theorized to be sensed/modulated at the brainstem level and smaller/weaker stimuli requiring cortical modulation as well (25–27). Given that all participants who aspirated silently on small liquid volumes had suffered cortical or subcortical lesions, it could be postulated that their ability to respond with a cough to smaller volumes invading the airway (i.e., weaker sensory stimulus) may had been compromised. In this early stroke phase, increased risk of silent aspiration (28), especially with smaller bolus volumes, has been previously noted as well (29); however, this hypothesis warrants further investigation and validation with a larger sample of silent aspirators.

Interestingly, maximum pitch elevation (measured acoustically) did not significantly correlate with most measures of laryngeal elevation, anterior hyoid excursion, or pharyngeal residue in our patients. This finding may relate to the fact that the neural control of these components stems from other cranial nerves and/or other branches of the vagus (and not the SLN). Another potential explanation relates to the scales used to rate these components (3- to 5-point scales), which may have been too crude to capture the patients’ performance. Using objective kinematic measurements would be important in order to fully understand whether maximum pitch elevation may be associated with other physiological markers of dysphagia, and should be explored in the future.

Finally, perceptual maximum pitch ratings performed by medical SLPs are sensitive in identifying patients with reduced maximum pitch. This finding is also in accordance with the findings of Malandraki et al. (18). An explanation offered by these authors relates to the binary scale used (normal vs. abnormal) to complete the pitch ratings, which appeared to be simple and significantly correlate with the acoustic measure of max *F*_0_. The clinicians providing these ratings also expressed that the scale and instructions were easy to follow. We acknowledge, however, that acoustic analysis is more accurate and thus more reliable. With the advent of readily available technological applications for acoustic analysis in smartphones, iPads, and computers, it is likely that this process can be easily implemented at the bedside. However, the validity and reliability of these apps needs to be examined before implementation.

Although the sample size was adequate for the overall analysis, the group of silent aspirators was small and further large-scale investigation is warranted. In addition, this study focused on analyzing the MBSImP variables that were neurophysiologically or biomechanically linked to vocal pitch elevation. We acknowledge that kinematic measures of hyolaryngeal excursion and upper esophageal sphincter opening would be important in future analysis in order to further understand the relationship between maximum pitch and swallowing biomechanics. Finally, we did not perform objective voice evaluations (e.g., stroboscopy) and cannot conclusively exclude the possibility of a laryngeal pathology in our sample. However, the detailed voice history collected at the beginning of the study did not reveal the presence of coexisting laryngeal or respiratory pathologies for any of the patients.

## Conclusion

The present investigation is the first to demonstrate that reduced maximum vocal pitch elevation measured acoustically is predictive of silent aspiration of small volume liquids with high sensitivity and moderate specificity in a sample of stroke patients early post stroke (<1 month post). Although this finding is promising, future large-scale studies focusing on further validating this finding and exploring its value as part of a dysphagia screening or clinical evaluation protocol are warranted.

## Ethics Statement

This study was carried out in accordance with the recommendations of the Institutional Review Boards of Teachers College, Columbia University and the JFK Rehabilitation Institute. All subjects gave written informed consent in accordance with the Declaration of Helsinki. The protocol was approved by the Institutional Review Boards of Teachers College, Columbia University and the JFK Rehabilitation Institute.

## Author Contributions

AR was instrumental in data collection, analysis, data interpretation, and manuscript preparation. KS and CZ contributed in subject recruitment, data collection, and manuscript review. MT contributed in data analysis, interpretation, and manuscript preparation. JBM contributed in data analysis and manuscript review. GM conceptualized the study and was instrumental in setting up the study design, data collection, data interpretation, and manuscript preparation. All authors have provided final approval of the version submitted and have agreed to be accountable for all aspects of the work performed.

## Conflict of Interest Statement

The authors declare that the research was conducted in the absence of any commercial or financial relationships that could be construed as a potential conflict of interest.

## Abbreviations

SLP, speech-language pathologist; CSE, clinical swallowing evaluation; VFSS, videofluoroscopic swallowing study; FEES, flexible endoscopic evaluation of swallowing; PAS, Penetration/Aspiration Scale; MBSImP, Modified Barium Swallow Impairment Profile; PES, pharyngoesophageal segment; SLN, superior laryngeal nerve; Max *F*_0_, maximum fundamental frequency; ROC, receiver operating characteristics; AUC, area under the curve.
