# Supplementary material for: Reduced Maximum Pitch Elevation Predicts Silent Aspiration of Small Liquid Volumes in Stroke Patients
Source: Front Neurol. 2017 Aug 25;8:436. doi: 10.3389/fneur.2017.00436 (PMC5574871; doi:10.3389/fneur.2017.00436)
Supplement: Supplementary file 1 [file table_1.docx]

Supplementary Material

Title: Reduced Maximum Pitch Elevation Predicts Silent Aspiration of Small Liquid Volumes in Stroke Patients

Authors: Akila Rajappa, Kristie R. Soriano, Courtney Ziemer, Michelle S. Troche, Jaime Bauer Malandraki and Georgia A. Malandraki^*^

*** Correspondence:** Georgia A. Malandraki; [malandraki@purdue.edu](mailto:malandraki@purdue.edu)

# Supplementary Tables

**Table A**. Penetration-Aspiration Scale (PAS) groupings used for ROC analysis based on the 8-point PAS developed by Rosenbek, Robbins, Roecker, Coyle, & Woods (1996)

| **Score** | **Description of Events** | **PAS Groupings for ROC Analysis** | | | |
| --- | --- | --- | --- | --- | --- |
| 1 | Material does not enter airway | 1 – 2: participants with normal swallowing | 1 – 5: Participants with normal or mild changes in swallowing (only penetration) |  | 1 – 7: Participants with all degrees of penetration or aspiration, except silent aspiration |
| 2 | Material enters the airway, remains above the vocal folds, and is expelled from the airway |  |  |  |  |
| 3 | Material enters the airway, remains above the vocal folds, and is not expelled from the airway | 3 – 8: participants with all degrees of penetration and aspiration |  | 3 – 5: Participants with penetration |  |
| 4 | Material enters the airway, contacts the vocal folds, and is expelled from the airway |  |  |  |  |
| 5 | Material enters the airway, contacts the vocal folds, and is not expelled from the airway |  |  |  |  |
| 6 | Material enters the airway, passes below the vocal folds, and is expelled into the larynx or out of the airway |  | 6 – 8: Participants with all degrees of aspiration | 6 – 8: Participants with all degrees of aspiration |  |
| 7 | Material enters the airway, passes below the vocal folds, and is not expelled from the trachea *despite effort* |  |  |  |  |
| 8 | Material enters the airway, passes below the vocal folds, and no effort is made to expel the material (silent aspiration) |  |  |  | 8: Participants with silent aspiration |

**Table B.** Detailed sample demographics and stroke specific characteristics

| **Patient** | **Age** | **Sex** | **Primary Diagnosis/Primary Side of Lesion** | **Stroke Onset (Days)** | **Type of Stroke** | **Areas affected** |
| --- | --- | --- | --- | --- | --- | --- |
| 1. | 66 | M | R-CVA | 3 | Hemorrhagic | R-Frontal, Temporal-parietal, Occipital, Basal ganglia |
| 2. | 43 | M | R-CVA | 2 | Ischemic | R-Basal ganglia |
| 3. | 77 | M | L-CVA | 3 | Ischemic | L-Temporal-occipital |
| 4. | 87 | M | L-CVA | 6 | Ischemic | L-Frontal, Temporal-parietal |
| 5. | 91 | F | R-CVA | 7 | Ischemic | R-Frontal, Temporal-parietal |
| 6. | 87 | F | R-CVA | 4 | Ischemic | R-Basal ganglia |
| 7. | 76 | F | R-CVA | 25 | Ischemic | R-Frontal |
| 8. | 53 | F | R-CVA | 20 | Hemorrhagic | R-Pons |
| 9. | 44 | F | R-CVA | 6 | Ischemic | R-Pons; Bilateral Thalamus |
| 10. | 68 | M | R-CVA | 4 | Hemorrhagic | R-Parietal, Temporal; L-Cerebellar |
| 11. | 69 | F | R-CVA | 15 | Ischemic | R-Thalamus |
| 12. | 85 | F | R-CVA | 15 | Hemorrhagic | R-Frontal, Temporal-parietal |
| 13. | 74 | F | L-CVA | 7 | Ischemic | L-Corona radiata, Internal capsule |
| 14. | 75 | F | R-CVA | 7 | Ischemic | R-Thalamus |
| 15. | 68 | M | L-CVA | 13 | Ischemic | L- Medulla |
| 16. | 52 | M | R-CVA | 14 | Ischemic | R-Thalamus, R-Midbrain |
| 17. | 75 | F | R-CVA | 20 | Hemorrhagic | R-Temporal-parietal, L- Cerebellum |
| 18. | 60 | M | R-CVA | 5 | Hemorrhagic | R- Basal ganglia, Corona radiata; L-Medulla |
| 19. | 85 | F | R-CVA | 10 | Hemorrhagic | R- Parietal, Basal ganglia |
| 20. | 67 | F | Bilateral CVA | 7 | Hemorrhagic | R- Parietal; L-Thalamus |
| 21. | 74 | M | R-CVA | 14 | Hemorrhagic | R-Frontal, Basal Ganglia, Temporal |
| 22. | 82 | M | R-CVA | 22 | Ischemic | R-Occipital, Parietal, Thalamus |
| 23. | 54 | F | R-CVA | 10 | Hemorrhagic | R-Thalamus |
| 24. | 90 | M | R-CVA | 14 | Ischemic | R-Pons |
| 25. | 88 | F | R-CVA | 11 | Ischemic | R-Occipital, Frontal |
| 26. | 65 | M | R-CVA | 19 | Hemorrhagic | R- Fronto-temporal |
| 27. | 94 | F | L-CVA | 18 | Ischemic | L-Thalamus |
| 28. | 84 | F | Bilateral CVA | 16 | Ischemic | Bilateral Pons |
| 29. | 54 | F | Bilateral CVA | 14 | Ischemic | R-Pons; L-Frontal white matter, Internal capsule, Pons, Cerebellum, Thalamus |
| 30. | 66 | M | Bilateral CVA | 19 | Ischemic | R-Frontal-Parietal; Bilateral Occipital, L- Corona radiata |
| 31. | 82 | F | L-CVA | 11 | Ischemic | L-Basal ganglia, Periventricular white matter |
| 32. | 60 | F | Bilateral CVA | 10 | Ischemic | R-Parietal, Temporal; L-Cerebellar |
| 33. | 91 | F | Bilateral CVA | 18 | Ischemic | R-Caudate Head, Occipital, Parietal; L-Cerebellum |
| 34. | 67 | M | Bilateral CVA | 18 | Ischemic | R-Basal Ganglia, Corona radiata; L-Occipital |
| 35. | 61 | M | L-CVA | 7 | Ischemic | L-Corona radiata |
| 36. | 58 | F | R-CVA | 7 | Ischemic | R-Frontal-parietal, Subcortical white matter |
| 37. | 87 | M | L-CVA | 16 | Ischemic | L-Frontal, Temporo-parietal |
| 38. | 60 | M | R-CVA | 15 | Ischemic | R-Brainstem, Midbrain, Central gyrus, Pons |
| 39. | 57 | M | L-CVA | 6 | Hemorrhagic | L-Corona radiata, Basal ganglia, Putamen |
| 40. | 80 | M | L-CVA | 9 | Ischemic | L-Fronto-Parietal, Corona Radiata, Thalamus, White matter |
| 41. | 53 | M | L-CVA | 14 | Ischemic | L-Corpus Callosum; R-Midbrain |
| 42. | 79 | M | R-CVA | 6 | Ischemic | R-Parietal, Periventricular white matter |
| 43. | 80 | M | R-CVA | 7 | Ischemic | R-Parietal |
| 44. | 84 | F | L-CVA | 6 | Ischemic | L-Cerebellum, Frontal |
| 45. | 68 | F | Bilateral CVA | 14 | Hemorrhagic | R-Parietal, L-Frontal |

*R-right, L-left; *CVA-Cerebrovascular Disease*

**
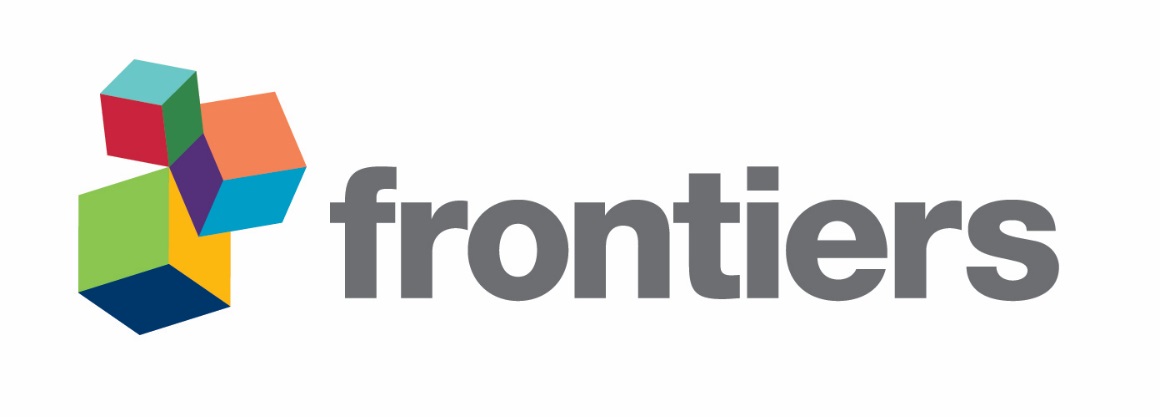
**
